# Economic Activities, Illness Pattern and Utilisation of Health Care Facilities in the Rural Population of Kwazulu-Natal, South Africa

**DOI:** 10.4102/phcfm.v1i1.24

**Published:** 2009-06-10

**Authors:** Monjurul Hoque

**Affiliations:** 1 Empangeni Hospital, South Africa

**Keywords:** determinants of health condition, economic activity, illness, utilisation, rural South Africa

## Abstract

**Background:**

The study was undertaken among the rural and black communities of the Uthungulu health district of the KwaZulu-Natal province, South Africa.

**Method:**

A cross-sectional community-based descriptive study was conducted. A multi-stage sampling strategy was adopted to obtain a representative sample of the communities.

**Results:**

The mean age of the population was 27 years and majority was female (54%). Among the adult population only 30% were educated, 19% were engaged in some form of economic activities while 9% were in the formal employment sector. The average monthly income per household was R1 301 (95% CI, R1 283; R1 308). The illnesses were reported by 27% of the total population over a period of one month. Notably higher rates of female individuals (29%) were sick compared to males (24%, p < 0.001). The rates of illnesses among adult females (39%) were also significantly higher than among males (31%, p < 0.009). Most of them (69%) attended primary health care (PHC) clinics for medical services, while 67% reported chronic conditions. Age (OR = 1.4), gender (OR = 0.711), education (OR = 0.64) and economic activities (OR = 1.9) were found to be associated with being ill or not.

**Conclusion:**

The rural black communities are underdeveloped and deprived, which results in higher prevalence of illnesses; however, the utilisation of PHC facilities is comparatively higher than in the rest of the province and other parts of the country. Interventions to improve community health care services among the deprived population should be focused through public health strategies such as all-encompassing PHC that includes health promotion, education and basic essential amenities.

## INTRODUCTION

South Africa is presently experiencing a quadruple burden of disease comprising of pre-transitional diseases such as chronic diseases or diseases of lifestyle, newly emerging cases of HIV/AIDS, other communicable conditions and injuries.^1^ There are links between the political history of South Africa and the social and natural environments. Apartheid policies, which restricted the political rights of nonwhite people, also limited their ability to attain better socio-economic and health status.^2, 3^ Women were predominantly affected as they had the lowest status of all.^4^ This resulted in the majority of the population being trapped into poverty. In the past, most South Africans did not have ample incomes or access to land and therefore could not afford their own housing.^2^ Before democratisation****, health inequalities in South Africa resulted in different patterns of health conditions and service utilisation among racial groups. Those highly affected were black people, particularly those who lived in rural areas.^5–7^ A high proportion of the vulnerable population was women and children.^5, 8^ Utilisation of preventive health care services was very low in South Africa. A study found that among South African children, 48.8% of Africans, 47.1% of Coloureds, 56.5% of Indians and 56.2% of Whites used a Road to Health Card and completion of vaccinations in the African population was lower than in other demographics, e.g. 70.5% of Africans had been vaccinated against TB compared to 95.6% of Coloureds, 93.5% of Indians and 92% of White people.^5, 9^

In 1994, against the international trend, South Africa suspended user fees for pregnant and lactating women as well as children aged under six years to improve right of access to public health care for the most vulnerable groups of the population. In 1997 user fees for all kinds of primary health care (PHC) services for the total population were suspended. Following the introduction of these policies, it was found that there was an increase in attendance patterns at health care facilities by rural communities.^10, 11^ While there was little or no increase in attendance of preventive services such as antenatal care, childhood immunisation and growth monitoring, the attendance of curative services increased by 77%.^10^

Woman living in rural areas present with an increased risk of ill health and AIDS-related deaths.^12, 13^ With the projected increase in the burden of the HIV epidemic in South Africa and in particular to the province of KwaZulu-Natal (KZN), it is essential that their health care needs are addressed through the PHC system to reduce the burden of demand for services at higher levels.^13^ On the other hand, economic inactivity (defined as people who are unemployed, unable to work, looked after a home, or retired) was found to be significantly associated with an increased risk of ill health in several studies.^14–16^

In South Africa, Demographic and Health Surveys (SADHS) are conducted every five years to measure different health indicators. The results are reported on national, provincial, urban as well as non-urban levels and this may mask the very high or low rates at a local (district) level.^11, 18^ However, all types of health problems that the rural population are suffering from and their socio-demographic determinants are not known. Therefore, this study aims to establish household demographics (to notice and depict different interactions), socio-economic activities, types of health problems, determinants of ill health and health care utilisation patterns in order to strategise public health interventions.

## METHOD

### Settings and population

The study was undertaken among the rural and black communities of the Uthungulu health district (one of the eleven districts) of KZN in South Africa with a residency of over 450 000 people. According to the census of 2001, nearly 90% of its (Uthungulu) people are rural, black and speak the local language (isiZulu), 75% are without any income, 30% have access to some form of piped water and are considered poor. The average monthly income per household is R661. Over half of the inhabitants of the area have no schooling or have some primary education. The Uthungulu health district is situated in the eastern and northern part of the province. Some parts are on the coastline of the Indian Ocean and the district lies approximately 170 km north of the commercial capital and the largest port city of Durban. There are two hospitals and 14 PHC clinics run by public sectors, which largely cover the rural and poorer section of the district. There are also two private hospitals and approximately 30 general practitioners. However, such services are urban-based and essentially within the means of the richer section of the district. Within the PHC facilities, there is a general shortage of doctors, nurses and other professional staff, e.g. pharmacists and radiographers. The public sector has appointed 462 community health workers (CHWs) from within the communities to cover more than 90% of households and to provide a wide range of services, including health promotion and education, to support communities in identifying health problems, to refer sick individuals to health care facilities and to provide treatment support for tuberculosis (TB) and HIV/AIDS. Each CHW (male or female) is allocated approximately 100 (range 90–110) households for their routine activities. They work 40 hours a week (Monday to Friday) and usually visit each household twice a month.

### Study design, sampling and data collection

A cross-sectional community-based descriptive study was conducted among the black rural population of the district. A total of 248 households were selected using multi-stage sampling techniques to cover rural parts of the district. At the first stage, 31 communities were randomly selected from a total of 462 communities (obtained from the district office). In the second stage, eight households from each selected community were selected by means of systematic random sampling techniques using the identity number of households used by the CHWs. In May 2005, the head or person responsible for child health care from each household was interviewed in his/her native language by CHWs. 31 CHWs were designated from the respective communities identified by random selection to conduct fieldwork (data collection). The head or health care giver of the household was requested to participate. The participation was voluntary in nature. Data was collected on all members of households. The residing members of the households were asked about their age, gender, education, occupation, types of activities they were undertaking, total family income, types of illnesses and uses of health care facilities in the preceding 30 days of the interviews. When an individual suffered from a sudden onset of illness for a shorter (< 15 days) period of time, it was then considered an acute type of illness (e.g. influenza, diarrhoea). On the other hand, it was considered as a chronic condition if the suffering period was longer (> 15 days).

The Empangeni Hospital Policy and Ethics Committee gave authorisation to conduct the study. The data was collected with the individuals’ informed consent. Full confidentiality and individual rights were maintained. No name of any participant or household was used in the presentation of data.

### Data analysis

Data were analysed using SPSS 12.0.1 for Windows Version 2003. The demographics and baseline outcome variables (both primary and secondary) were recapitulated using descriptive summary measures: expressed as mean (standard deviation) or median (minimum-maximum) for continuous variables and percentages for categorical variables. Chi-square tests of association were performed using two-sided tests at the 0.05 level of significance between two categorical variables. The binary logistic regression method was used to find the significant predictor for outcome variables. For regression models, the results were expressed as odds ratios, corresponding two-sided 95% confidence intervals and associated p-values. P-values were reported to three decimal places with values less than 0.001 reported as < 0.001.

## RESULTS

### Demographic information

Data were collected from 236 households that consist of 1 299 residents. This resulted in an average of 5.5 people per household. Four households were excluded due to their unwillingness to take part and/or absence of the qualifying respondents. Eight households’ data were incomplete and therefore rejected, which resulted in the response rate of 95%. Over half of the households (58%) had members who were living elsewhere – totaling an amount of 260. A total of 55 (36 female and 19 male) were receiving some form of social grant (mainly a disability grant). Grants were defined as alimony, compensation for injuries and state pensions.

The average age of the population was 27 years, of which the majority (54%) were female (Table 1). Almost one-third of the total population was younger than 15 years old (31%). Gender was equally distributed among the population up to the age of 29 years. A higher rate of females (22%) was observed compared to males (14%) over the age of 30 years (c^2^ =17, p < 0.009). 27% of the females were of reproductive age (15–44 years).

**TABLE 1:** Age and gender distribution of 1254 people living in 236 households

	GENDER		
		
AGE GROUP	FEMALE (%)	MALE (%)	TOTAL (%)
0 – 9 yrs	8.4	8.3	16.7
10 – 19 yrs	13.3	13.7	27.0
20– 29 yrs	10.1	9.4	19.5
30– 39 yrs	7.3	4.2	11.5
40– 49 yrs	6.3	4.3	10.6
50– 59 yrs	2.9	2.6	5.5
60 yrs and above	5.9	3.2	9.1

Among the adult population (age 15 years and over) of 882 (68% of the total population), only 30% had some education and 19% were engaged in any form of economic activities, but only 9% were in formal employment, another 9% (male and female equally) were involved in local gardening, and 1% in local trading. A significantly higher proportion of females (59%) was engaged in household economic activities (p < 0.001). The average earning by the residing members of households per month was R860 (95% CI, R737.31; R994.69). The total monthly average income per household was R1 301 (95% CI, R1 283.74; R1 308.26). This included the contribution made by the people staying outside the households.

### Health conditions and utilisation of health care facilities

A total of 455 people (35% of the total of 1 299) attended the health care facilities or visited the practitioners over a period of one month, of which 72 (60 female and 12 male) attended for preventive and promotive care (antennal care, pap smear, immunisation, voluntary counselling and testing of HIV and family planning). The actual illness (self-reported) was reported by 373 (27% of the total population), of which a higher proportion was female (57%). Among the female population the proportion of illness was 29% and the rate of illness among males was 24%. This difference was statistically significant (p < 0.001). Similarly, the rates of adult female and male (age over 15 years) illnesses were 39% and 31% respectively and the difference was statistically significant (p < 0.009). Most individuals (69%) attended PHC clinics for their conditions (illness and preventive services), and 25% attended public hospitals. Among those who were ill, most (67%) reported chronic conditions, e.g. high blood pressure, TB, diabetes and arthritis (see Table 2). The commonly reported acute conditions were influenza, diarrhoea and trauma.

**TABLE 2:** The self-reported health conditions and attendance to health care facilities of 455 individuals according to gender over a one-month period

REASONS FOR ATTENDING HEALTH CARE FACILITIES	FEMALE (% OF TOTAL ILLNESS)	MALE (% OF TOTAL ILLNESS)	TOTAL (%)
**PROMOTIVE AND PREVENTIVE CARE**			
Antenatal care	8.6	0	8.5
Family planning	0.9	0	0.9
Immunisation	2.7	2.7	5.4
Pap smear	1.3	0	1.3
**CHRONIC CONDITIONS**			
Arthritis	2.7	1.1	3.8
Asthma	1.8	0.7	2.5
Diabetes	6.1	2.2	8.3
Diarrhoea	3.4	2.0	5.4
Eye conditions	1.8	2.9	4.7
HIV-related diseases	3.8	2.7	6.5
Hypertension	9.0	7.1	6.1
TB	5.6	6.3	11.9
Skin conditions	0.2	0.4	0.6
Others	1.8	3.4	5.2

Other medical problems included poisoning, dog bites, chicken pox, abortions, dental problems, ear problems, renal diseases, malaria, measles, rape cases, snake bites and stroke. Of the ill individuals, 11% were admitted to hospitals. This translated to a rate of 3% of the total study population (95% CI: 2%; 4%) admitted to hospital over a period of one month.

Among the ill individuals, 37.7% had visited the health care facilities once and over 25% had visited them more than three times over a month. The number of health care facility visits by the people was associated with their age (p = 0.002). Results of logistic regression showed that the age of the individual was a significant predictor (OR = 1.40) for reporting an illness (Table 3). Males were less likely to report illness than females (OR = 0.711). Educated (> 5 years of schooling) people were also less likely to report illness (OR = 0.674). The economically active population (adults) tends to use health care facilities more than the younger group when they are sick (OR = 1.9).

**TABLE 3:** Output of logistic regression analysis for reporting an illness

VARIABLES	B	SIG.	ODDS RATIO (OR)	95.0% C.I. FOR OR (LOWER; UPPER)
**Age group**				
Age	0.642	0.004	1.40	(1.255; 1.758)
Gender (male)	-0.352	0.006	0.703	(0.547; 0.904)
Education > 5 yrs	-0.395	0.013	0.674	(0.493; 0.921)
Not economically active population	1.247	.001	3.598	(2.651; 8.882)
Constant	-.226	.364	.798	

Variable(s) entered in step 1: age group (0–5 years as reference group), gender, education, not economically active population

## DISCUSSION

This is a cross-sectional population-based descriptive study that aimed to establish the socio-demographic profile, economic activities at household level, prevalence of health conditions and utilisation of health care facilities by the rural population of the Uthungulu health district of KZN. The study revealed a high rate of 27% overall, with 39% of adult females and 31% of adult males in the rural Uthungulu district reporting illness over a period of one month. This figure is higher than the findings from the SADHS 2003, where 15% adult males and 24% adult females reported ill and attended health care facilities (one month preceding the surveys).18 The results also indicate that older people (age over 45 years) were more likely to become ill and attend health care facilities, similar to other findings.^19^

The population of this rural district is young and poor. Onethird of the household population is younger than 15 years. The age and gender distribution of the study population is similar to the population distribution of the district (Census 2001) and the non-urban population sample of SADHS 2003, indicating that there is probably minimum selection bias in our study.^18^ Conversely, the results of this study represent the rural population of the Uthungulu district only. The male population over the age of 30 years shows dramatic reduction (14%) compared to females (23%), indicating a smaller number of males in the rural households. There are two possible reasons for this: migration out of the district to work outside and a decrease in the adult male population due to death. Both reasons could be applicable, as high numbers of young males are seen to die due to the high burden of the HIV and TB epidemic in South Africa.^20, 21^ It is imperative to note that the average number of people per household of 5.5 is higher than the findings of SADHS 2003 (only four in non-urban areas).18 The people from these rural households who have migrated to urban areas are still upholding supportive relationships with the rural households. This is related to rural cultural practices in this part of the country where people have extended families as a way of traditional living. Only 19% of the adult population from the rural communities are involved in some form of economic activity (gardening, local trading, formal/informal employment, etc.), where females were found to be leading the households. The overall socio-economic conditions therefore signify that the rural communities of the district are poor and underdeveloped. The number of recipients of any social grant was low (4%) compared to the figures of KZN and nationally (8.8% and 10.9% respectively). This lower rate of social grant recipients is probably related to the low level of awareness and unavailability of the services to this rural population. It is also found that the adult active population who were not working were 3.6 times (OR = 3.598) more likely to become ill than those who were working. In order to improve the health and overall socio-economic conditions, individual, familial or communal involvement (formal or informal or both) in gardening or farming could be commenced through interdepartmental collaboration as a part of the holistic public health intervention.

The prevalence rates of illness for the overall population (27%), namely 31% of adult males and 39% of females, are higher compared to the findings of SADHS 2003.^18^ Nevertheless, the overall illness rate and illness according to age groups are similar to other African countries.^15^ The higher rate of women reporting illness must be viewed with regard to the increasing mortality rate among young women in KZN.^22^ Therefore, this trend of more women being ill is likely. The finding that most individuals are reporting chronic conditions is in line with the findings and prediction of escalating demands of chronic diseases and their risk factors in developing countries.^22^ It is encouraging to see that virtually all (97%) of the ill individuals from this rural communities attended services in the formal health sector. This is similar to the findings from an earlier study from another rural district.^23^ This trend of a higher rate of public sector service utilisation is different from the rest of the province or country, as shown in SADHS 1998 and 2003.^11, 18^ The higher rate of utilisation of public health care facilities is related to the economic availability of these services, unavailability of private sector services (e.g. GPs and private hospitals) and the quality of health care service delivery in public health care facilities (which warrants further studies). Since the unemployment rate is higher, the low rate of involvement in any economic activities resulted in poor socio-economic conditions, making the individuals reliant on free public sector services. This is confirmed with the higher utilisation of PHC facilities (69%). Therefore, the higher rate of illnesses and utilisation of public health care facilities indicate the need for present and future health care facilities in the district. Males are less likely to become sick than females (OR = 0.711) and educated people are also less likely to become sick (OR = 0.674). These findings concur with the trends found elsewhere.^23–25^ It might be the case that educated people are more aware of their rights to use and benefit from new health information and technologies.

### Limitations

Recall bias is likely as the recall of illness of all household members is gathered from the head of the household or the individual responsible for child health care. However, studies have showed that such bias is limited if the gap between the time of being ill and information collection is short (e.g. one week or one month).^22^ Data collected by non-professionals from the households on the types of illness were not based on actual medical conditions as health care professionals (nurses or doctors) would have diagnosed them based on the standard classification of diseases (e.g. International Classification of Primary Care – ICPC-2), and this resulted in a lack of comparability with other findings. However, the prevalence of illness, illness pattern and its determinants are of great value to a particular population in the health planning sector.

### Conclusion

The high levels of socio-economic dispossessions among the rural population of the Uthungulu health district are evident, with a higher prevalence of illnesses than the rest of the province and country. This is comparable to other poorer regions of sub-Saharan Africa. The vast majority of the population attend PHC facilities when they are ill and the utilisation rate is resultantly higher. The main socio-economic determinants of health are age, gender, level of education and economic activities. As a consequence, interventions to improve community health should focus on improving the education levels of the population, while the provision of basic essential amenities is also crucial. Health planners should allocate indispensable resources to areas where the educational level of the population is poor. This also warrants a vital endeavour to introduce the provision of comprehensive PHC services (e.g. health promotion and education and voluntary counselling and testing) and to target all issues related to socioeconomic development at large in this rural population.
